# Self-assembly of dental surface nanofilaments and remineralisation by SnF_2_ and CPP-ACP nanocomplexes

**DOI:** 10.1038/s41598-018-37580-w

**Published:** 2019-02-04

**Authors:** James R. Fernando, Peiyan Shen, Christina P. C. Sim, Yu-Yen Chen, Glenn D. Walker, Yi Yuan, Coralie Reynolds, David P. Stanton, Colin M. MacRae, Eric C. Reynolds

**Affiliations:** 1 Oral Health Cooperative Research Centre, Melbourne Dental School, Bio21 Institute, The University of Melbourne, Melbourne, Victoria, Australia; 2 Microbeam Laboratory, CSIRO Mineral Resources, Clayton, Victoria, Australia

## Abstract

Dental caries, erosion and hypersensitivity are major public health problems. SnF_2_ is used widely in oral care products to help prevent/treat these conditions. Casein phosphopeptide-stabilised amorphous calcium phosphate nanocomplexes (CPP-ACP) are a biomimetic nanotechnology of salivary phosphopeptide-ACP complexes that deliver bioavailable calcium and phosphate ions to promote dental remineralisation (repair). We show here using *in vitro* studies and a double-blind, randomised controlled, cross-over design *in situ* clinical trial that SnF_2_ and CPP-ACP interact to form a nanofilament coating on the tooth surface and that together they are superior in their ability to promote dental remineralisation. Sn(II) by cross-linking the CPP-ACP helps to stabilise the complexes which improves delivery to the tooth surface and enhances binding and ion incorporation into tooth mineral. The combination of SnF_2_ and CPP-ACP in oral care products may significantly improve their efficacy in prevention/treatment of dental caries/erosion and hypersensitivity.

## Introduction

The global prevalence of dental caries, erosion and hypersensitivity have increased over the last two decades^1–3^. This has led to increased interest in developing better treatments and preventive regimes for these conditions. Dental caries is a multifactorial lifestyle-associated bacterial disease that results in the progressive dissolution and loss of tooth mineral, termed demineralisation^4^. Demineralisation of the enamel and dentine crystals, idealised as hydroxyapatite Ca_10_(PO_4_)_6_OH_2_^5^ occurs due to a state of undersaturation with respect to that mineral in the surrounding fluid following organic acid production by adjacent plaque bacteria through dietary sugar fermentation (caries) or by direct dietary food acid exposure (erosion). The demineralisation process is opposed by inherent repair mechanisms through bioavailable calcium and phosphate ions obtained from saliva^6,7^ that rebuild the apatite crystals in a process termed remineralisation^8^. Frequent consumption of dietary sugar and/or food acids can overcome the salivary protective effect to produce ‘net’ demineralisation, increasing the porosity and structural weakness of the tooth. This process eventually leads to lesion cavitation and the requirement for restorative intervention. However, demineralised lesions that have progressed to be clinically visible but not yet cavitated can still be repaired through remineralisation. As saliva is limited in its remineralisation potential^6,7^, remineralisation aids have been developed to increase remineralisation intraorally to treat and help prevent dental caries and erosion^9^. Fluoride, usually as sodium fluoride is a well recognised remineralisation agent that promotes remineralisation of caries and erosion lesions when released into saliva from oral care products^7^. Fluoride ions drive remineralisation together with bioavailable saliva calcium and phosphate ions to form fluorhydroxyapatite [Ca_10_(PO_4_)_6_(OH)_2−2×_F_2×_]^10^. However, even in those with normal saliva flow, levels of bioavailable calcium and phosphate ions in saliva can become limiting for remineralisation. This has resulted in new technologies utilised in novel oral care products that provide bioavailable calcium and phosphate ions for remineralisation^10^.

Casein phosphopeptides (CPP) are known to stabilise calcium, phosphate and fluoride ions allowing a high supersaturation of soluble ions in saliva for remineralisation of mineral deficient tooth structure^11^. The binding motif within the CPP contains phosphoserine and glutamic acid in a cluster sequence similar to the salivary protein statherin, hence the CPP are a biomimetic of statherin. The binding motif -Ser(P)-Ser(P)-Ser(P)-Glu-Glu- attracts calcium ions and subsequently phosphate and fluoride ions to form soluble nanocomplexes of amorphous ion clusters, referred to as CPP-ACP or CPP-ACFP^12^. Stabilisation of these ions in electroneutral nanocomplexes allows their delivery to deeper parts of the lesion so that lesion fluid supersaturation and remineralisation occurs throughout the lesion body^13^. These nanocomplexes have been incorporated into oral care and professional dental products that have been shown to improve remineralisation efficacy^11^.

Recently, there has been renewed interest in stannous fluoride (SnF_2_) due to results suggesting that the stannous ions as well as the fluoride ions are effective in inhibiting acid-induced demineralisation of enamel and dentine^14–16^. It has been hypothesised that Sn(II) interacts with the tooth surface to increase hardness and prevent exposure of calcium on the enamel surface to increase its resistance to demineralisation^17–19^.

The interaction of CPP-ACP and SnF_2_ has not been investigated. Here we demonstrate that Sn(II) stabilises CPP-ACFP complexes to enhance remineralisation and fluoride uptake and to promote the formation of a nanocoating on the tooth surface.

## Results and Discussion

### Enamel remineralisation

Initially enamel remineralisation was tested *in vitro* using two treatments at pH 5.6. These were 1: 0.4% w/v CPP-ACP + 220 ppm F as SnF_2_ + 70 ppm F as NaF (CPP-ACP + SnF_2_) and 2: 0.4% w/v CPP-ACP + 290 ppm F as NaF (CPP-ACP + NaF). Human enamel blocks containing demineralised lesions were prepared according to a previous protocol^13^. The enamel blocks were incubated with fresh remineralisation solution every 48 hours for 10 days. Remineralisation of the enamel lesions was measured using transverse microradiography^13^ (TMR). Treated lesions were also analysed using scanning electron microscope electron dispersive x-ray spectroscopy (SEM-EDS).

The *in vitro* remineralisation model demonstrated the combined CPP-ACP + SnF_2_ treatment was more effective at remineralising enamel subsurface lesions than CPP-ACP + NaF despite both solutions having the same CPP-ACP and fluoride concentrations. The extent of remineralisation with the CPP-ACP + SnF_2_ treatment was 32% greater than with the CPP-ACP + NaF treatment (see Table 1).

**Table 1:** Comparison of enamel subsurface lesion parameters before and after remineralisation *in vitro* (pH 5.6) and *in situ* as measured by TMR.

	Treatment	LDd (µm)^a^	ΔLD (µm)^b^	∆Zd (vol% min.µm)^c^	∆Zd-∆Zr (vol% min.µm)^d^	%Remin^e^
*In vitro*	CPP-ACP + NaF	124.6 ± 18.6	23.5 ± 12.8	3587.0 ± 923.9	1259.8 ± 370.8^A^	35.0 ± 5.4^A^
	CPP-ACP + SnF_2_	127.4 ± 20.5	30.2 ± 17.6	3784.6 ± 1398.7	1757.1 ± 757.8^A^	46.1 ± 5.8^A^
	**p-value** ^§^	NS > 0.05	NS > 0.05	NS > 0.05	<0.05	<0.0001
*In situ*	NaF	103.6 ± 10.9	2.0 ± 3.5^abc^	2728.9 ± 578.8	291.4 ± 48.6^abc^	10.8 ± 0.8^abc^
	SnF_2_	97.6 ± 6.9	5.0 ± 3.8^de^	2273.3 ± 294.5	245.4 ± 43.4^def^	10.8 ± 0.8^def^
	CPP-ACP	105.8 ± 6.9	10.8 ± 1.2^a^	2729.2 ± 427.6	367.2 ± 68.0^adgh^	13.4 ± 1.0^adgh^
	CPP-ACP + NaF	103.4 ± 8.1	12.5 ± 7.0^bd^	2742.4 ± 490.1	670.2 ± 102.8^begi^	24.6 ± 2.1^begi^
	CPP-ACP + SnF_2_	104.3 ± 6.3	15.0 ± 2.9^ce^	2527.5 ± 449.1	776.6 ± 159.9^cfhi^	30.6 ± 1.6^cfhi^
	**p-value** ^ǂ^	NS > 0.05	<0.0001	NS > 0.05	<0.0001	<0.0001

^a^LDd = lesion depth after demineralisation, ^b^ΔLD = reduction in lesion depth after remineralisation, ^c^ΔZd = integrated mineral loss prior to remineralisation, ^d^ΔZd-ΔZr = gain in mineral content after remineralisation; ^e^%R = percent remineralisation ((ΔZd-ΔZr/ΔZd) * 100%). Displayed as mean ± standard deviation. ^§ǂ^ANCOVA (α = 0.05) NS not significant. Differences between means were measured using *post hoc* multiple comparison tests on the marginal means using a Sidak adjustment: ^Aabcdefghi^Values in the same column similarly marked are significantly different (p < 0.05).

**Figure 1:**
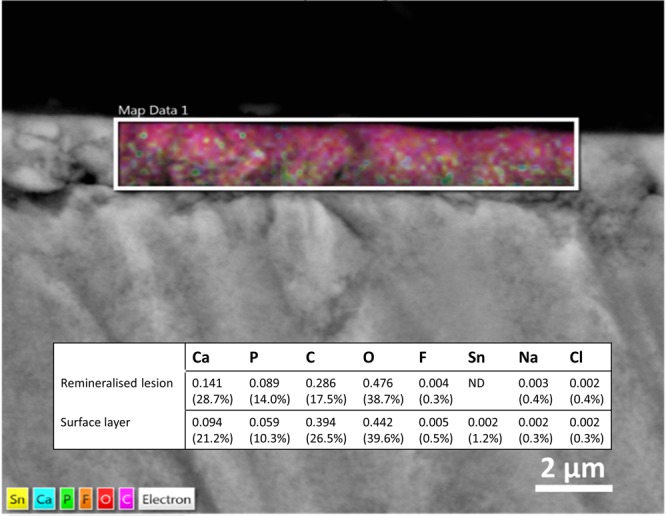
SEM-EDS analysis of enamel treated with CPP-ACP + SnF_2_ *in vitro*. The coloured map represents element distribution in the surface layer with the inset table expressing elemental composition of the highlighted area as mole proportion (weight percentage in parentheses). ND = not detected.

SEM-EDS analysis of the CPP-ACP + SnF_2_ treated enamel lesions revealed they had a calcium to phosphorous ratio of 1.58–1.59, consistent with apatite^20^, containing fluoride (see Fig. 1). The enamel had a mineralised surface layer rich in carbon (26.5%) as well as tin (1.3%). The detection of carbon within the surface layer (26.5%) suggested an organic component was bound to the mineral phase, most likely CPP upon release of cargo ions. This is in agreement with previous studies that showed CPP have a high affinity for apatite and regulate apatite growth^21^. Taking into account the oxygen of the phosphate in the surface layer, and the theoretical oxygen as hydroxide in stoichiometric HA, the resulting carbon to oxygen ratio of the surface layer was 2.1. This finding is in agreement with the observed surface layer in the subsequent dentine surface experiment.

Enamel remineralisation was also tested *in situ* using five treatments (mouthrinses) at pH 4.0. These were 1: 0.4% w/v CPP-ACP + 220 ppm F as SnF_2_ + 70 ppm F as NaF (CPP-ACP + SnF_2_), 2: 0.4% w/v CPP-ACP + 290 ppm F as NaF (CPP-ACP + NaF), 3: 0.4% w/v CPP-ACP, 4: 220 ppm F as SnF_2_ + 70 ppm F as NaF (SnF_2_) and 5: 290 ppm F as NaF. The *in situ* remineralisation study was conducted according to a previously published protocol^22,23^ using TMR to assess remineralisation. In addition electron probe microanalysis (EPMA) was used to assess the fluoride and tin contents of the lesions. The mean unstimulated and stimulated salivary flow rates for the participants of the study were 0.73 ± 0.33 mL/min and 3.00 ± 1.02 mL/min respectively. All participants completed the *in situ* trial with no reported adverse events.

The CPP-ACP + SnF_2_ mouthrinse produced the greatest remineralisation in the enamel subsurface lesions (30.6 ± 1.6%) and was significantly higher compared with all other treatments, including the CPP-ACP and CPP-ACP + NaF mouthrinses (see Table 1). The NaF and SnF_2_ mouthrinses were not statistically different to each other in terms of percentage remineralisation and produced the lowest mean remineralisation.

**Figure 2:**
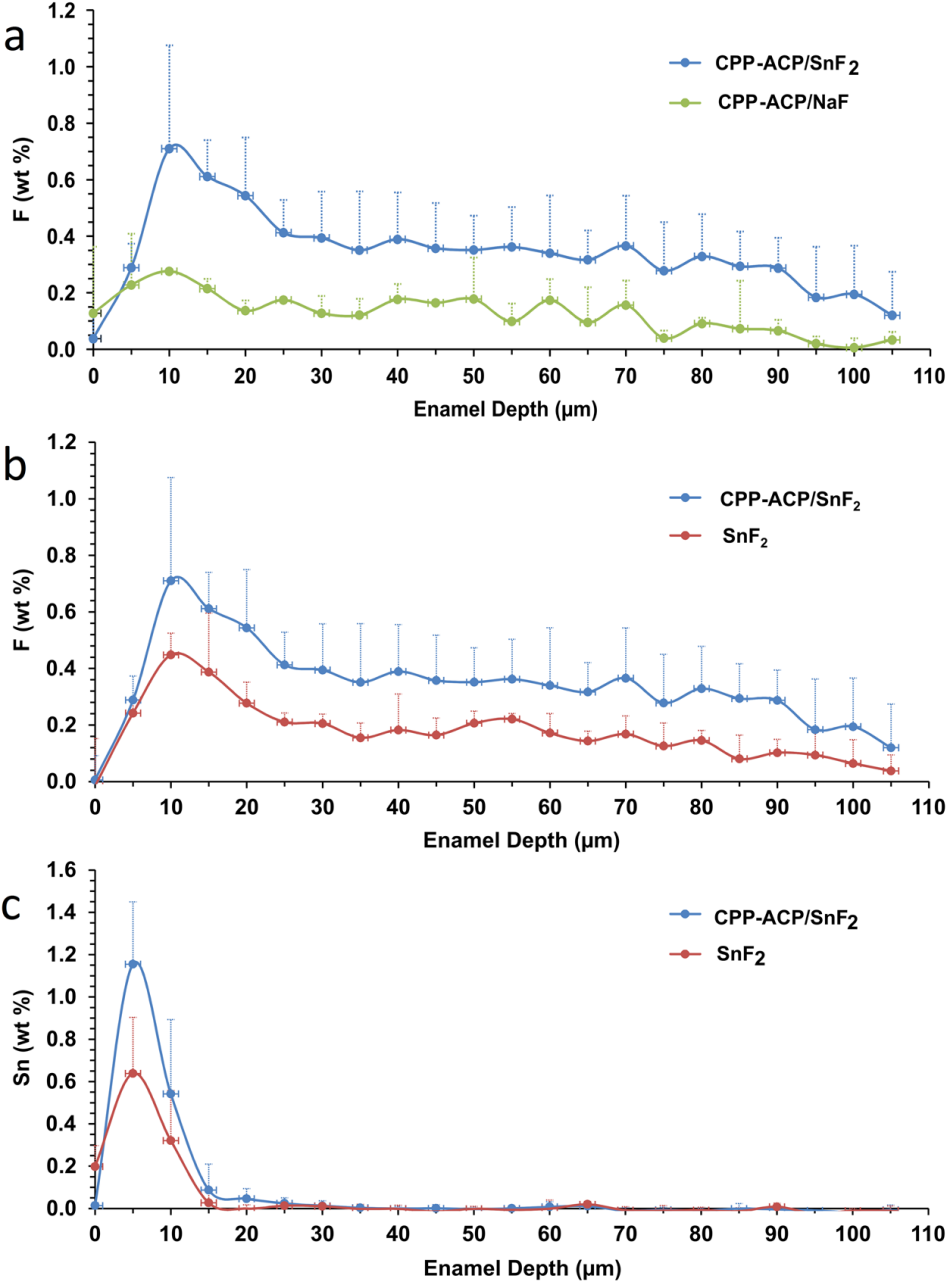
EPMA analysis of atomic weight percentage within enamel subsurface lesions treated *in situ*. **a** = fluoride weight % comparison between CPP-ACP + SnF_2_ and CPP-ACP + NaF treated lesions, **b** = fluoride weight % comparison between CPP-ACP + SnF_2_ and SnF_2_ treated lesions, **c** = stannous weight % comparison between CPP-ACP + SnF_2_ and SnF_2_ treated lesions.

Approximately twice as much fluoride was measured in lesions treated with CPP-ACP + SnF_2_ compared with the SnF_2_ treated lesions (see Fig. 2b). The fluoride content in the CPP-ACP + SnF_2_ treated lesions was also higher (throughout the lesion) than the CPP-ACP + NaF treated lesions, demonstrating the combination of CPP-ACP and SnF_2_ was more effective in promoting fluoride uptake when compared with CPP-ACP + NaF (see Fig. 2a). The tin content of the CPP-ACP + SnF_2_ treated lesions was approximately twice that of the lesions treated with SnF_2_ alone (see Fig. 2c). For both treatments the tin was mainly concentrated in the outer 10μm of the lesion, with the maximum tin content detected 5μm from the enamel surface.

Each treatment solution from the *in vitro* and *in situ* experiments was analysed for total and free ion concentrations to calculate the percentage of ions bound to CPP nanocomplexes (see Table 2). The majority of stannous ions were CPP-bound in the CPP-ACP + SnF_2_ solution: at pH 4.0, 90.8% of stannous ions were bound, and at pH 5.6, 99.3% were bound.

**Table 2:** Ion concentrations of calcium, phosphorus, fluoride and tin in treatment solutions at pH 5.6 and 4.0 as measured using AAS and ion chromatography.

	pH	Treatment	Ca mM	Pi mM	F mM	Sn mM
Total	5.6	0.4% CPP-ACP + SnF_2_	15.29 ± 0.17	10.88 ± 0.11	15.39 ± 0.09	5.58 ± 0.14
		0.4% CPP-ACP + NaF	14.56 ± 0.05	11.11 ± 0.17	15.0 ± 0.33	—
	4	0.4% CPP-ACP + SnF_2_	16.25 ± 0.16	11.62 ± 0.22	15.24 ± 0.63	5.67 ± 0.12
		0.4% CPP-ACP + NaF	15.13 ± 0.37	11.16 ± 0.20	14.8 ± 0.77	—
		0.4% CPP-ACP	15.00 ± 0.33	11.17 ± 0.42	—	—
CPP-bound	5.6	0.4% CPP-ACP + SnF_2_	15.25 (99.7%)	10.52 (96.7%)	6.33 (41.1%)	5.55 (99.3%)
		0.4% CPP-ACP + NaF	14.53 (99.8%)	7.20 (64.8%)	4.0 (26.8%)	—
	4	0.4% CPP-ACP + SnF_2_	12.53 (77.1%)	9.50 (81.8%)	8.64 (56.7%)	5.15 (90.8%)
		0.4% CPP-ACP + NaF	10.30 (68.1%)	2.89 (25.9%)	12.2 (82.7%)	—
		0.4% CPP-ACP	2.72 (18.1%)	2.45 (21.9%)	—	—
Free	5.6	0.4% CPP-ACP + SnF_2_	0.04 ± 0.00 (0.3%)	0.36 ± 0.01 (3.3%)	9.07 ± 0.14 (58.9%)	0.037 ± 0.00 (0.7%)
		0.4% CPP-ACP + NaF	0.03 ± 0.00 (0.2%)	3.91 ± 0.18 (35.2%)	11.0 ± 0.72 (73.2%)	—
	4	0.4% CPP-ACP + SnF_2_	3.72 ± 0.17 (22.9%)	2.12 ± 0.10 (18.2%)	6.59 ± 0.06 (43.3%)	0.52 ± 0.01 (9.2%)
		0.4% CPP-ACP + NaF	4.82 ± 0.20 (31.9%)	8.27 ± 0.26 (74.1%)	2.6 ± 0.02 (17.3%)	—
		0.4% CPP-ACP	12.28 ± 0.38 (81.9%)	8.72 ± 0.02 (78.1%)	—	—

Data are displayed as mean ± standard deviation. Percent of total ions CPP-bound and free indicated in parentheses.

It was evident from both the *in vitro* and *in situ* enamel experiments that the addition of SnF_2_ to CPP-ACP improved remineralisation by increasing the release of calcium phosphate and fluoride ions within the enamel lesions. The mechanism for the increased efficacy of the CPP-ACP + SnF_2_ treatment solutions was expounded by the ion analysis where the addition of SnF_2_ to CPP-ACP appeared to increase the binding capacity of CPP to stabilise larger soluble nanocomplexes. The CPP-bound calcium to CPP molar ratio (Ca:CPP) was an important marker of CPP efficacy as it strongly correlated with percent enamel lesion remineralisation *in situ* (R = 0.99). As each solution had a CPP concentration of 1.33 mM, at pH 4.0 the Ca:CPP molar ratios of the CPP-ACP, CPP-ACP + NaF, and CPP-ACP + SnF_2_ treatments were 2.0, 7.7, and 9.4 respectively and the %R values were 13.4 ± 1.0%, 24.6 ± 2.1% and 30.6 ± 1.6%, respectively (Table 1). While it has been reported that the addition of fluoride to CPP complexes increases their calcium binding capacity and remineralisation efficacy^13,24^, the current study is the first to show that tin acts in a similar manner.

### Dentine surface interaction

Human dentine specimens with exposed tubules were treated with three solutions *in vitro* and subsequently analysed using SEM and SEM-EDS. The treatment solutions were 1: 5% w/v CPP-ACP, 2: 500 ppm F as SnF_2_, and 3: 5% w/v CPP-ACP + 500 ppm F as SnF_2_ (CPP-ACP + SnF_2_). Untreated dentine was the negative control.

**Figure 3:**
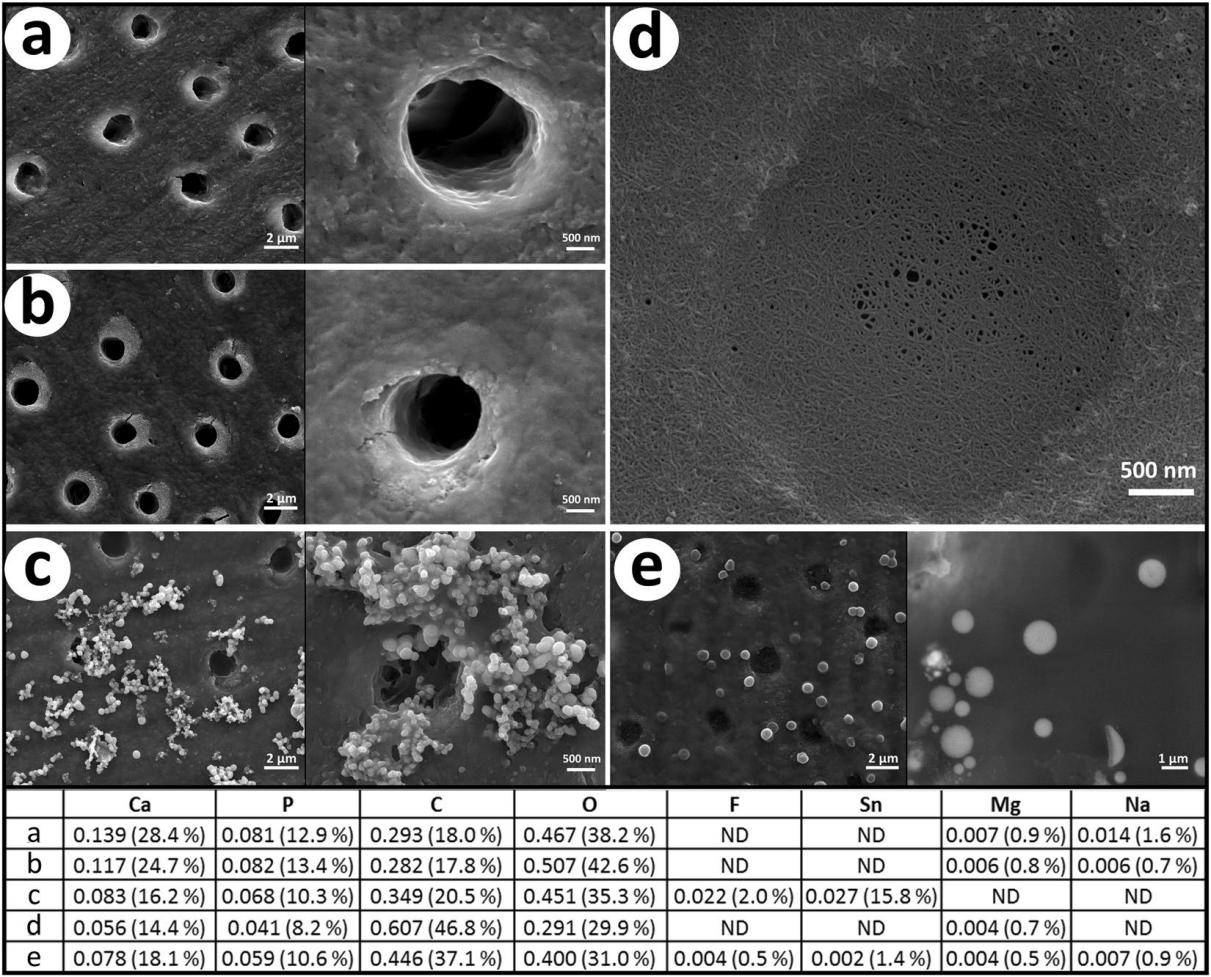
SEM images of (**a**) control dentine (**b**) CPP-ACP treated dentine (**c**) SnF_2_ treated dentine (**d**,**e**) CPP-ACP + SnF_2_ treated dentine. Inset tables display elemental composition as mole proportion (weight percentage in parentheses) from EDS measurements (C: EDS measurements of electron dense deposits D: EDS measurements of nanocoating E: EDS measurements of electron dense spheres).

Representative images from each dentine treatment are shown in Fig. 3. The control dentine showed a surface with no precipitation and patent tubules. Similarly, the dentine treated with only CPP-ACP appeared to be relatively unchanged. In contrast, the SnF_2_ treated dentine displayed globular deposits on the dentine surface and within the dentine tubules. The dentine treated with CPP-ACP combined SnF_2_ displayed areas with a visible coating of cross-linked nanofilaments covering the dentine (including the tubules) accompanied by electron dense spheres (see Fig. 3d,e). These spheres ranged from nanosized up to 2 µm in diameter.

The elemental analysis (wt%) of the dentine surface from the control group (a) revealed a composition consistent with the organic and inorganic components of sound dentine^25,26^. Dentine treated with CPP-ACP had a composition closely matching the control dentine.

The analysis of the surface deposits visible on the dentine treated by SnF_2_ alone revealed it contained on average 15.8 wt% tin and 2.0 wt% fluoride. Other elements present on the surface layer were oxygen (35.3 wt%), carbon (20.5 wt%), calcium (16.2 wt%) and phosphorous (10.3 wt%). The appearance of these surface deposits was similar to that seen in previous studies assessing the effect of SnF_2_ on surface dentine^27,28^. Ellingsen and Rolla^27^ hypothesised the surface mineral present on dentine after SnF_2_ treatment was likely to be a mixed phosphate-coated calcium fluoride (CaF_2_) deposit and a stannous phosphate derivative. Other authors have suggested Sn(OH)_2_, Sn_2_(PO_4_)OH, Ca(SnF_3_)_2_, Sn_3_F_3_PO_4_, Sn_2_(OH)PO_4_, Sn_3_F_3_PO_4_, or SnHPO_4_ may precipitate on the surface of dentine treated with SnF_2_^14^. As the interaction of SnF_2_ with dentine is complex and not fully understood, it was difficult to extrapolate the SEM-EDS observations to speculate which of these tin-containing phases may have been present on the dentine surface. The molar ratio of tin to fluoride, calculated from the wt% values as detected by SEM-EDS, was 1.26. This suggested more tin-containing phases were present on the surface than fluoride-containing phases and that additional fluoride was likely to have been present in the solution phase.

The electron dense spheres visible on the dentine treated with CPP-ACP + SnF_2_ were rich in oxygen and carbon (37.1 wt% and 31.0 wt%, respectively) as well as calcium (18.1 wt%), phosphorous (10.6 wt%), tin (1.4 wt%), sodium (0.9 wt%), magnesium (0.5 wt%) and fluoride (0.5 wt%). The nanofilament coating present on these samples was similar in composition although relatively higher in carbon content (46.8 wt% carbon, 29.9 wt% oxygen, 14.4 wt% calcium, 8.2 wt% phosphorous and 0.7 wt% magnesium) and did not contain detectable levels of fluoride or tin. The molar ratios for these elements are presented in Fig. 3.

The hydrodynamic radius of CPP-ACP complexes has been shown to be approximately 2 nm, with a slightly larger radius observed when fluoride is incorporated as CPP-ACFP^12,24,29^. The spherical particles observed following the CPP-ACP + SnF_2_ treatment varied in size. In some instances they were nanosized or up to 2 µm in diameter. The phenomenon observed on the dentine surface after this treatment and the structures of the spherical particles and nanofilament coating can be explained by the elemental composition obtained from SEM-EDS. The high carbon and oxygen percentage suggested the spherical particles had an organic component accompanying the bundles of calcium, phosphate, fluoride and stannous ions. Under the experimental conditions, this organic structure was consistent with CPP bound together or cross-linked by the electron dense atom tin. Using the major peptides present in the commercial CPP-ACP preparation as representative of the peptide content, it can be shown that the measured carbon to oxygen ratio of the sphere was very close to that of CPP. The major and representative CPP sequence β(1–25) has 117 carbon atoms, 59 oxygen atoms and 4 phosphorous atoms per peptide resulting in a carbon to oxygen ratio of 1.98^30^. Assuming the total carbon content of the sphere could be attributed to the CPP content and using the molar ratios shown in Fig. 3, the relative phosphorous present in the peptide (0.017 phosphorous) could be subtracted from the total mole proportion of phosphorous observed (0.059) to give the remaining proportion of phosphorous present as inorganic phosphate within the CPP-ACFP complex (0.042). The corresponding mole proportion of oxygen as inorganic phosphate within the CPP-ACFP complex (0.168) subtracted from the total mole fraction of oxygen observed then provided an estimate of the remaining oxygen contributed by the CPP (0.232). The carbon to oxygen ratio of the organic component could then be calculated as 1.92, which closely corresponds to the C: O molar ratio for the CPP; consistent with CPP being responsible for the carbon content of the sphere. Additionally, the calcium to CPP molar ratio was 18 and the molar ratio of CPP to stannous ion was 2. As CPP-ACFP nanocomplexes typically contain 15 calcium ions per peptide^24,29^, the finding from the current study is consistent with the incorporation of the stannous ions increasing the calcium binding capacity of CPP, which is similar to the result with the ion analyses in the enamel experiments.

The electron dense coating of cross-linked nanofilaments observed covering the dentine in the SEM images following treatment with CPP-ACP + SnF_2_ was especially high in carbon (46.8 wt%). Remarkably, the carbon to oxygen ratio of this layer (2.1) is identical to that of the organic component in the surface layer of the CPP-ACP + SnF_2_ *in vitro* treated enamel lesions (2.1). While this was slightly higher than the theoretical ratio for CPP, a cross-linking of CPPs on the surface through the well characterised chemically induced β-elimination of serine phosphate in casein would have resulted in some loss of oxygen explaining the higher carbon to oxygen ratio^31,32^. This finding is consistent with a network of cross-linked CPPs in nanofilaments being formed by stannous ion-promoted self-assembly on both enamel and dentine following treatment with stannous-containing CPP-ACFP solutions.

**Figure 4:**
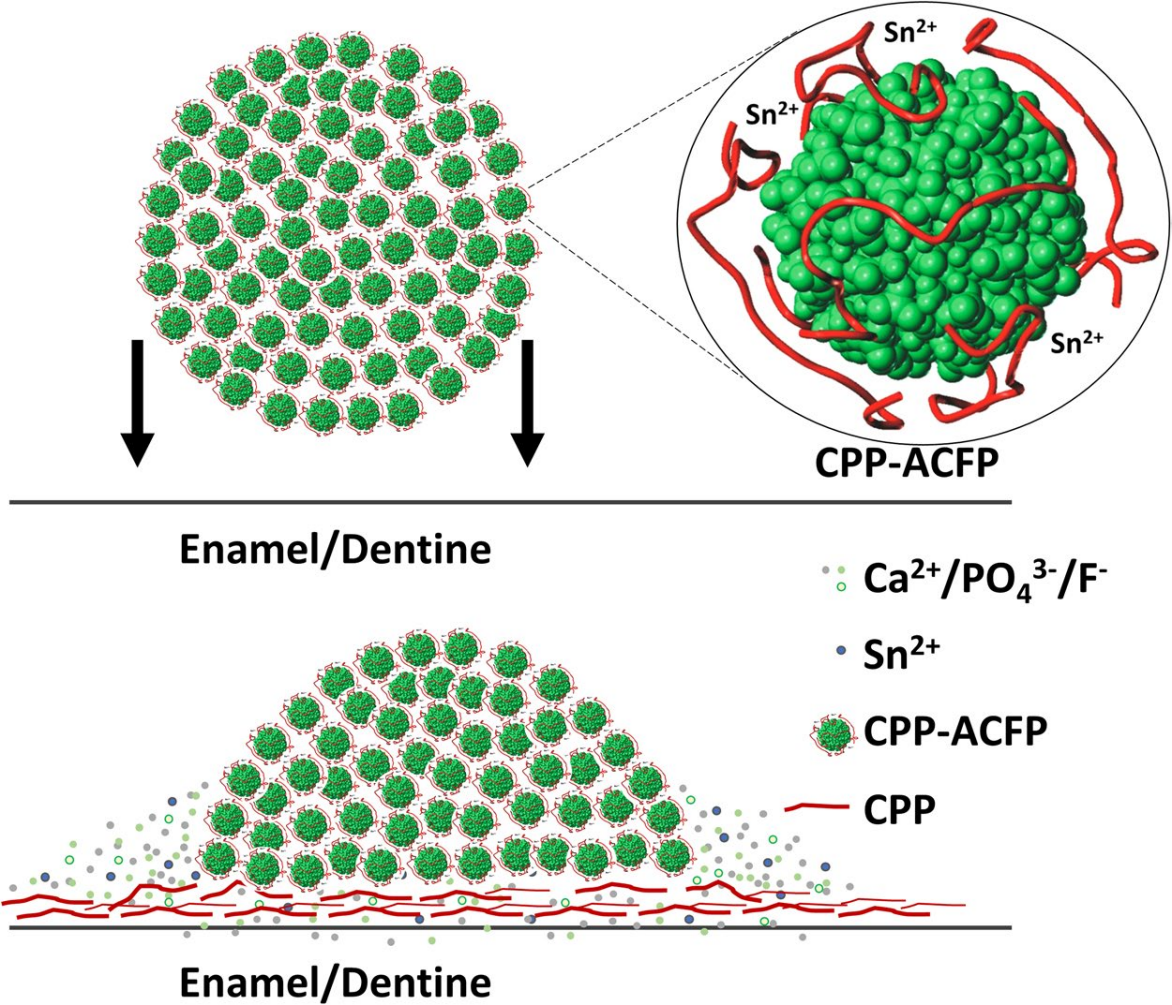
Diagram illustrating the proposed mechanism for Sn^2+^ mediated release of Ca^2+^/PO_4_^3−^/F^−^ from cross-linked CPP nanocomplexes and subsequent CPP nanofilament coating of the tooth surface.

To explain the tooth surface interaction of the CPP-ACP + SnF_2_ a schematic illustrating the mechanism is shown in Fig. 4. CPP-ACFP nanocomplexes^24^ are cross-linked by stannous ions to form larger spherical particles up to 2 µm in diameter as visible in the SEM images. Upon contact with the tooth surface the negatively charged residues of the CPP became increasingly attracted to the exposed positively charged apatite crystal faces on the surface. In addition, the stannous ions are attracted to the surface phosphate, promoting complex dissociation and CPP adsorption onto the surface while releasing the cargo of calcium, phosphate and fluoride ions contained within the cross-linked nanocomplexes. The CPP bind to surface calcium ions and the driving force for bonding is the formation of lower free-energy, polydentate structures^21^. The CPP complexes accordingly change conformation and cross-link on the tooth surface to form a cross-linked nanofilament coating, thereby releasing their payload of calcium, phosphate and fluoride to the tooth surface promoting remineralisation. The cross-linking of the CPP on the tooth surface would be facilitated by stannous-catalysed β-elimination of phosphoseryl residues to form reactive dehydroalanine residues that can engage in Michael addition reactions with nucleophiles of other CPP residues (e.g. Lys amines) to produce covalently cross-linked CPP nanofilaments^31,32^.

## Conclusions

Significantly higher enamel remineralisation was observed *in vitro* by the combined CPP-ACP + SnF_2_ solution compared with the CPP-ACP + NaF solution. A synergistic effect upon enamel remineralisation by CPP-ACP and SnF_2_ was observed *in situ* by the combined CPP-ACP + SnF_2_ solution which showed significantly higher remineralisation than the CPP-ACP + NaF, CPP-ACP, NaF and SnF_2_ solutions. The addition of SnF_2_ to CPP-ACP increased complex stability, ion binding capacity and ion delivery at the tooth surface for remineralisation. Combining CPP-ACP and SnF_2_ resulted in a unique interaction with the tooth surface. Stannous ions aggregated CPP nanocomplexes and promoted ion release and the self-assembly of cross-linked CPP nanofilaments upon contact with the tooth surface. In summary, the combination of CPP-ACP and SnF_2_ in oral care products may significantly improve their efficacy in prevention and treatment of dental caries, erosion and hypersensitivity.

## Materials and Methods

### Preparation of treatment solutions

A commercial CPP-ACP preparation was used to prepare the CPP-ACP-containing solutions (Recaldent™, Mondelez International AMEA Pte Ltd). Solutions were adjusted for pH using 1.0M hydrochloric acid. A pH of 4.0 was used for the *in situ* enamel remineralisation solutions as the SnF_2_ solution alone was not stable in water with a pH higher than 4.0. When preparing solutions for the enamel remineralisation *in vitro*, the addition of SnF_2_, NaF and CPP-ACP to the desired concentration yielded an unadjusted pH of 5.6; accordingly the CPP-ACP + NaF *in vitro* solution was adjusted to this pH to allow comparisons at the same pH value. It is estimated that toothpaste is diluted 1:4 in saliva during toothbrushing^33^. Therefore, the concentrations of fluoride and CPP-ACP in the fluoride-containing *in vitro* and *in situ* remineralisation solutions were 290 ppm and 0.4% (w/v), respectively, to simulate a 1:4 dilution of toothpaste containing 1450 ppm F or 2% (w/v) CPP-ACP. The ratio of SnF_2_ to NaF in the CPP-containing enamel treatment solutions was formulated to mimic a commercial SnF_2_/NaF-containing OTC dentifrice (Oral B®, Procter and Gamble). A higher concentration of CPP-ACP and fluoride was used for the *in vitro* dentine treatment solutions to simulate placing a commercial dental professional product containing CPP-ACP (Tooth Mousse®, GC Corporation) directly on an exposed tooth root *in vivo*. All solutions prepared were stable at room temperature.

### Enamel remineralisation protocol

For the *in vitro* enamel remineralisation model, 24 extracted human third molars were sectioned into enamel blocks and demineralised to produce artificial carious lesions, using a modified protocol to that described by White^34^. Enamel blocks were each sectioned into an experimental half-block and control half-block. Each group of experimental half-blocks (n = 12) was suspended in the respective remineralisation solutions for 10 days at 37 °C with a change of solution every 48 hours. Subsequently, the half-blocks were paired with their control half-block and analysed for mineral content change using transverse microradiography (TMR) as described previously^13^.

The *in situ* clinical study had a randomised, controlled, double-blind cross-over study design. The University of Melbourne Human Research Ethics Committee approved the research conducted in this study (Approval Number 1441572) and the work was carried out in accordance with the Code of Ethics of the World Medical Association (Declaration of Helsinki). The clinical trial registration number is ISRCTN 10532332 and date of registration is 23^rd^ November 2018. Artificially demineralised carious lesions were created on enamel blocks sectioned from human third molars and the blocks were each cut into experimental and control half-blocks as in the *in vitro* remineralisation model. Experimental half-blocks were inserted into custom-made palatal appliances to create a plaque retention site over the enamel lesions as described previously^10,23^. Each appliance contained four enamel half-blocks with subsurface lesions, two on each side of the appliance^10,23^. It was calculated that eight participants were required to provide the required statistical power (90%, p < 0.05) based on previous investigations using a similar cross-over *in situ* model^10,35,36^. The required sample size was calculated using the G*Power Version 3.1 sample size package^37^ and was based on a repeated measures analysis of variance with five levels, an effect size of 0.97, a correlation, ρ, between any pair of treatment means of 0.5 and a non-sphericity correction ε of 0.5. The effect size of 0.97 was based on detecting differences between ΔZd-ΔZr means of 70 (CPP-ACP + NaF and CPP-ACP + SnF_2_) and a common standard deviation of 100 within groups. The non-sphericity correction adjusts for heterogeneity in the variances of the repeated measures. With a 5% significance level and a power of 90% at least six subjects were required. To allow for subject attrition eight subjects were recruited for the study.

Eight healthy adults (average age 43 ± 11 years; four males and four females) living in Melbourne, Australia with a fluoridated (0.9  ppm F), reticulated water supply participated in this double-blind, randomised, cross-over study. The participants were recruited from staff and students of the University of Melbourne and provided informed, written consent to participate in the study. Study inclusion criteria were: age 18–60 years; at least 22 natural teeth; unstimulated whole salivary flow rate of ≥0.2 ml/min and chewing gum-stimulated whole salivary flow rate ≥1.0 ml/min. Study exclusion criteria included: currently using antibiotics or medications that may affect salivary flow rates or a history of severe oral disease. The trial was conducted at the Royal Dental Hospital of Melbourne in 2014. Each participant wore their custom-made palatal appliance containing the four enamel half-blocks with subsurface lesions and rinsed with 5 mL of one of the five treatment solutions for one minute, four times each day for 14 consecutive days (treatment period) as described previously^10^. Participants were randomly assigned to each rinse. Each participant was assigned a number by the biostatistician (GDW) and randomisation was effected using a standard randomisation table for the five coded rinses. Participants were instructed to continue their normal dietary regime during the study and were given a toothbrush and sodium fluoride toothpaste (1450  ppm F) to brush their teeth twice a day. The intra-oral appliances were removed during oral hygiene procedures and were kept in a sealed humidified container. Participants were also instructed to clean their intra-oral appliances with a toothbrush and a fluoride-free denture paste supplied to them, taking care to avoid the attached enamel half-blocks. At the conclusion of the 14 days, participants rested from the study for one week (washout period) then began another treatment with another randomly assigned treatment solution. This was repeated until participants had rinsed with all five solutions. During the treatment periods subjects maintained a diary recording each rinse and duration of rinse. Following the treatment period, participants returned their appliance, empty rinse tubes and diary to the investigators and new enamel half-blocks were attached for the next treatment period. At the completion each experimental half-block was paired with its control half-block following the remineralisation period for analysis of mineral change using TMR and Sn and F levels using electron probe micro-analysis (EPMA). Researchers and participants were blind to the treatment code. A senior staff member held the treatment code securely which was only released after data collection and analysis.

Initial lesion depth (LDd), lesion depth change (LDd-LDr), initial mineral content (ΔZd), mineral content change (ΔZd-ΔZr), and percent remineralisation (%R) were measured from analysis of the demineralised and remineralised lesion mineral profiles from the TMR images as described previously by Cochrane *et al*.^23^ For the *in vitro* data, a two-sample t-test was used to measure differences in lesion parameters (LDd, LDd-LDr, ΔZd, ΔZd-ΔZr and %R) between the two treatments. For the *in situ* data, the subject was the unit of analysis and the same lesion parameters were compared across the five treatments using analysis of covariance (ANCOVA). The primary outcome measure was integrated mineral gain/loss (ΔZd-ΔZr and %R) determined by TMR and the secondary outcome was Sn and F wt% enamel uptake measures determined using EPMA. Data were analysed for normality using Q-Q plots and the Shapiro-Wilk test and homogeneity of variance was tested using Levene’s test^38^. *Post hoc* pairwise differences between treatments were performed on the estimated marginal means using the Sidak adjustment for multiple comparisons. The statistical significance was set at p < 0.05. SPSS software version 22 (IBM Corp. NY, USA) was used for all statistical tests.

### Analysis of ion concentrations in the enamel treatment solutions

The total and free (not stabilised within CPP complexes) calcium, tin, phosphate and fluoride concentrations were calculated from the 0.4% CPP-ACP + SnF_2_ + NaF solutions at both pH 4.0 and 5.6 and the 0.4% CPP-ACP solution at pH 4.0. The total ion concentration was measured by diluting 1 mL of solution with 19 mL of 1.0M HNO_3_ and left for 24 hours before being centrifuged at 1000 g for 15 minutes at room temperature. The supernatant was analysed for calcium and tin using atomic absorption spectroscopy (AAS), as well as phosphate and fluoride using ion chromatography (Dionex™, Thermo Fisher Scientific). The ‘free’ ion concentration was calculated by filtering a sample of solution in a centricon using a 1000 MWCO filter (Pall Corporation) and the resulting filtrate was centrifuged at 3000 g for 60 minutes at room temperature. The supernatant was then analysed for ion concentration using AAS and ion chromatography as described for the total ion concentration measurements.

### Scanning electron microscope energy-dispersive x-ray spectroscopy (SEM-EDS) analysis of enamel

SEM-EDS analysis was conducted on TMR sections of enamel lesions treated *in vitro* by CPP-ACP + SnF_2_ to assess the distribution of elements within the lesion following remineralisation. The samples were examined at 10 kV under low vacuum using a solid-state diode backscatter electron detector in a FEI Quanta FEG 200 SEM operating at 10 kV with an energy-dispersive spectrometer (Bio21 Advanced Microscopy Facility, Victoria, Australia). Characteristic x-rays from areas of interest were then detected using an energy dispersive x-ray spectrometer and microanalysis software (AZtec Microanalysis Suite Ver 3.1, Oxford Instruments).

### Electron probe micro-analysis (EPMA) analysis of enamel

TMR sections from the CPP-ACP + SnF_2_, CPP-ACP + NaF and SnF_2_ *in situ* treatments were chosen for elemental analysis using EPMA. The tooth sections within the slides were embedded in epoxy resin (Epofix™, Struers, Denmark) on a specimen holder. The embedded enamel sections were initially polished using 2,400 grit abrasive paper, then were polished using three and one µm diamond polishing pastes until finally optical smoothness was achieved with a 0.25 µm aluminium oxide polishing paste. Samples and standards were coated with a 12–15 nm layer of carbon using a BOC Edwards Auto 306 Coater Evaporator coater. The electron probe (JXA-8500F HyperProbe JEOL, Japan) was operated at 15 kV accelerating voltage, 10 nA beam current, the beam was defocused to a spot of 5 µm to minimise induced damage following the methodology of Cochrane *et al*.^39^ Lesions were analysed with a 5 µm step between each analysis point. The elements measured were Ca, P, O, Na, Mg, Cl and F. Oxygen was measured by integrating across the peak for 40 s and background of 20 s was measured separately. Counting times for the peak and background were Ca (10 s), P (10 s), Na (10 s), Mg (10 s), Cl (8 s), and Sn (20 s). The standards were Sylvite (KCl), natural (Wilberforce) Fluorapatite (Ca_5_(PO_4_)_3_F), Halite (NaCl), Spinel (MgAl_2_O_4_) and Cassiterite (SnO_2_).

Quantitative line scans were collected across the lesions starting from sound enamel and traversing into the epoxy resin perpendicular to the surface layer. The two sigma detection limits for each of the elements were Ca 320, P 2000, O 2800, Na 400, Mg 210, Sn 300, Cl 350 and F 1300  ppm. Data were matrix corrected using a ϕ(ρZ)-parabolic method correction procedure implemented in STRATAGEM 3.0 thickness and compositional thin film analysis package, (SAMx, Saint Andre de la Roche, France, 1997). Fluorescence by the characteristic lines and by the continuum were corrected using this method for each x-ray line.

### Dentine surface treatment

Twenty extracted human third molars were sectioned into twenty one mm thick discs using a water-cooled diamond saw. To remove the smear layer, the discs were exposed to 15% EDTA for two minutes^40^ after which the discs were rinsed thoroughly with filtered deionised water (Milli-Q®, Millipore Corporation) for five seconds and blotted dry. The discs were placed into a sealed humidified environment before exposure to experimental solutions. Discs were randomly allocated into four groups with the negative control group having two discs and the three treatment groups having six discs each. The groups of discs were exposed to either no treatment (negative control), or 10 mL of 5% CPP-ACP, 500 ppm F as SnF_2_, or 5% CPP-ACP with 500 ppm F as SnF_2_ for 20 minutes before being removed, immersed in filtered deionised water for five seconds and stored in a humidified environment.

### SEM/SEM-EDS analysis of dentine

One disc from the control group and four discs from each of the experimental groups were desiccated with silica gel for 72 hours. Following dehydration, the discs were mounted on sample holders, gold sputter-coated (2 nm) and examined with an Everhart-Thornley detector in a FEI Quanta FEG 200 SEM at 10 kV under high vacuum (Bio21 Advanced Microscopy Facility, Victoria, Australia). The remaining discs from each group were desiccated using silica gel for 72 hours and examined using SEM-EDS as described for the enamel samples.
